# Dr. Charles A. Kingsbury

**Published:** 1892-06

**Authors:** C. A. Brackett, E. N. Harris

**Affiliations:** President; Corresponding Secretary


### Obituary.

### DR. CHARLES A. KINGSBURY.

At the monthly meeting of the American Academy of Dental Science, held in Boston March 2, 1892, the following resolutions were adopted:

WHEREAS, Charles A. Kingsbury, M.D., D.D.S., late of Philadelphia, an associate member of the American Academy of Dental Science, has recently been called away from these earthly scenes into the higher existence beyond the grave, we, his fellow members, would offer the following resolutions of respect to his memory.

*Resolved*, That the Academy has received with sincere sorrow the intelligence of the decease of our highly-esteemed friend and associate.

*Resolved*, That, in the death of Dr. Kingsbury, the profession has lost one of its brightest and most honored members, and the city and community in which he lived one of its most exemplary and useful citizens. Dr. Kingsbury was engaged in active practice and Professor of Dental Histology and Operative Dentistry in the Philadelphia Dental College through a long series of years, and contributed much to elevate and adorn his profession. He lived to an advanced age, and had the pleasant satisfaction, in his declining years, of seeing the science and art of dentistry, and the standing of the profession, far advanced towards the ideal that he had formed for it in the thoughts and aspirations of his earlier years.

*Resolved*, That we tender our sympathy and condolence to his surviving family in their heavy bereavement and sorrow, and the Corresponding Secretary be requested to forward a copy of these resolutions to them, and that a copy be entered upon the records of the Academy, and published in the dental journals.

C. A. BRACKETT, D.M.D., *President.*
E. N. HARRIS, D.D.S., *Corresponding Secretary.*

